# A Semantic Web for bioinformatics: goals, tools, systems, applications

**DOI:** 10.1186/1471-2105-9-S4-S1

**Published:** 2008-04-25

**Authors:** Nicola Cannata, Michael Schröder, Roberto Marangoni, Paolo Romano

**Affiliations:** 1 Department of Mathematics and Computer Science, University of Camerino, Camerino (MC), I-62032, Italy; 2 Biotechnology Centre, TU Dresden, Dresden, D-01307, Germany; 3 Computer Science Department, University of Pisa, Pisa, I-56127, Italy; 4 Bioinformatics, National Cancer Research Institute, Genova, I-16132, Italy

## Introduction

Network Tools and Applications in Biology (NETTAB) [1] is a series of workshops focused on the most promising and innovative Information and Communication Technologies (ICT) tools and to their usefulness in Bioinformatics. These workshops aim at introducing participants to innovative network standards and technologies that are being applied to the biology field. To this end, each year a special emphasis is given to a focus theme. Workshops also include special sessions devoted both to the general theme of the series of workshops, i.e. “Network Tools and Applications in Biology”, and to further topics selected by local organizers.

Biological data integration issues were already discussed in previous editions of this series of workshops, including topics such as “CORBA and XML: towards a bioinformatics integrated network environment” (NETTAB 2001) [2], “Agents in Bioinformatics” (NETTAB 2002) [3], “Workflows management: new abilities for the biological information overflow” (NETTAB 2005) [4] and “Distributed Applications, Web Services, Tools and GRID Infrastructures for Bioinformatics” (NETTAB 2006) [5,6].

The Seventh NETTAB workshop was held at the Computer Science Department of the University of Pisa, on June 12-15, 2007, having “A Semantic Web for Bioinformatics: Goals, Tools, Systems, Applications” as focus theme. Adjunct themes were “Algorithms in bioinformatics” and “Formal Methods for Systems Biology”.

This *BMC Bioinformatics* Supplement includes the best papers and posters – representing all the themes - from works presented at the workshop.

## A Semantic Web for bioinformatics

### Motivation for the focus theme

The quantity of biological information is increasing at an impressive rate. An integrated access to this huge amount of information requires complex search and retrieval software and automation of analysis processes. Automation of integration procedures mainly concerns how to link data, how to select and extract information and how to pipe retrieval and analysis steps. This automated approach to data analysis requires the adoption of new technologies and tools in the bioinformatics domain.

Some reference points have already been assessed, or are emerging, towards this goal: the adoption of XML schemas for information models specification, the definition of XML based languages for data representation and exchange, the implementation of Web Services for automated access to analysis tools and data, the creation of computerised pipelines and workflows for the definition and the execution of basic and complex analysis [7]. Workflow enactment portals can bring added value, allowing also non-expert researchers to take profit from automated procedures.

However, while these first steps towards data integration and processes automation have been made, little has been made for supporting semantic integration. What is needed are shared definitions of knowledge domains, i.e. ontologies, association of biological concepts to existing data, metadata information describing information sources and search tools able to make the best use of this additional information. Databases and tools should be made available on the Web, or better on the Semantic Web [8], that is the evolution of the World Wide Web permitting this information to be understandable and usable by software agents (i.e. autonomous, reactive and proactive computer systems) [9].

The definition of ontologies and their application to software and database tools may be seen as a first, needed attempt to organize the information, overcoming heterogeneity of data structures. But the problem of associating the information sources and the huge amount of data with concepts defined in these ontologies is a big one. The addition of semantic contents in current databases would give an essential contribution to the best integration of distributed biological information.

The development of metadata for biological information, on the basis of Semantic Web standards, and its definition for all information sources can also be seen as a promising approach for a semantic based integration of biological information.

### Meeting structure

The Opening Lecture, entitled “Pathway Commons: A public library of biological pathways on the Semantic Web”, was given by Gary Bader, University of Toronto. This lecture was selected with the idea of offering an overview of the problems faced by bioinformaticians while developing new data integration tools, in a not yet semantic era, and the perspectives of adopting Semantic Web technologies.

Sessions devoted to the focus theme aimed at getting together biologists, bioinformaticians, computer scientists and linguists trying to understand usefulness of a Semantic Web for bioinformatics, its possible goals, most promising standards, technologies and tools, with the final objective of devising which bioinformatics research problems can be solved by the Semantic Web and which are the short, medium and long term perspectives in applying Semantic Web technologies to bioinformatics.

In the first session, the aims and perspectives for the development of a Semantic Web for bioinformatics were discussed. It included an invited lecture by Eric Neumann, founder and co-chair of the W3C Semantic Web Healthcare and Life Science Interest Group (HCLSIG) [10]. The W3C HCLSIG is bringing together industry leaders and academic researchers to identify domain-specific applications that will benefit from Semantic Web technologies [11]. Topics of his talk included the vision for the communities, the HCLS group's scope and participants, and its current activities.

The second session discussed Semantic Web technologies and tools. Two invited lectures were respectively given by Antoine Isaac, University of Amsterdam, and Olivier Bodenreider, National Library of Medicine. Isaac presented ongoing activities within the W3C Semantic Web Deployment (SWD) Working group [12], that is currently working on a recommendation for the Simple Knowledge Organization System (SKOS) [13], which is intended to simplify the RDF/OWL representation of ontologies. Bodenreider reviewed bio-ontologies (see also [14]), and their central role in the Semantic Web (“Bio-ontologies: The cream in the Semantic Web layer cake” was the title of his lecture).

The third session focused on applications. A joint invited lecture was given by Michael Schroeder, Biotec TU Dresden, Albert Burger, Heriot-Watt University, and Robert Stevens, University of Manchester, who introduced Sealife, a Semantic Grid Browser for the Life Sciences [15].

Both sessions devoted to adjunct themes, selected by local organizers, had renowned invited speakers from the University of Pisa. The session on “Algorithms in bioinformatics” was opened by Fabrizio Luccio, who presented, in a homonymous talk, a global historic survey of the relationships between Information Theory and Biology. The session entitled “Formal Methods in Systems Biology” hosted an invited talk by Pierpaolo Degano, who discussed problems and perspectives of the application of formal languages to the description of biological systems.

The NETTAB 2007 web site includes almost all presentations that were given at the workshop [16].

### Panel discussion

Besides outlining the promising features of the Semantic Web in bioinformatics, the workshop also intended to support as much discussion as possible through open discussions and, especially, a final panel discussion on “Goals and perspectives of a Semantic Web for Bioinformatics” that was participated by invited speakers and chairs.

From the discussion, it emerged that the promises of the Semantic Web can really be of a paramount importance for bioinformatics, but undoubtedly there is still a lot to do. The current phase can still be considered as a pioneer one, in which scientists are getting familiar and becoming aware of the possibilities and possible scenarios that are offered by this new concept. Furthermore, related technologies still need to be improved and adapted or tuned. Recommendations should be provided by the World-Wide Web Consortium (W3C) and successful examples could be spurred by the HCLSIG.

Controlled vocabularies and ontological frameworks already acquired a wide diffusion in biomedical sciences. Although, during the discussion, it was reinforced that the HCLSIG has not the scope to develop them, there are other groups and institutions that can support development, and effectively are developing, biomedical ontologies, like the National Center for Biomedical Ontology (NCBO) [17]. Now, one of the main issues consists in bridging them.

Actually, scientists should be urged to expose their data and should be instructed on how to present these to the world, and on how to identify and represent them. Data sharing in the community was restated to be a major necessity. In a first approximation, data can also be kept as they are, while semantic layers and links can be built upon them by the community itself. Semantics and the Semantic Web have been explicitly recognized as “complicated”, while end users would like to have friendly tools and to find everything “on their desktop”.

Other important concerns about the data, coming also from the audience, were trust and provenance and, in general, transparency.

An interesting observation was that now semantics is actually embedded in the software that manages and analyses the data. In order to facilitate the advent of the Semantic Web, this knowledge should be removed from the code and put just in the data contents, therefore shifting from “intelligence in the software” to “intelligence in the data”.

The take-home message from the workshop was that “we are not there yet [18], but still on the way”. Some good building blocks have been developed and some successful experiences are showing the way, but some further mechanisms to facilitate things are still necessary (e.g. some technologies to support friendly insertion of semantics in web pages). Then, it will be possible to go beyond web navigation. Thanks to semantics interconnection and interlinking, ontology driven browsing will finally be achieved.

## Summary of best contributions

After a selective review process, performed by the Program Committee and some external reviewers, twelve articles have been accepted for publication in this Supplement to *BMC Bioinformatics*. These papers are extended and improved versions of the best oral presentations and posters of the NETTAB 2007 workshop. In the following paragraphs, we briefly review them. The complete proceedings of the workshop are also available [19].

### A Semantic Web for bioinformatics

The session on Semantic Web tools and applications is represented in this Supplement by five contributions.

Ontologies are one of the pillars on which the Semantic Web vision is built. The paper by Alexopoulou *et al *[20] concerns ontologies construction, which still critically suffers from the lack of widely accepted methodologies and automatic construction tools. Due to the huge amount of academic publications, a very important area in biomedical research is text mining. Automatic term recognition methods are applied by the authors of this paper with the aim of automatically deriving lists of terms and relations between them. An experiment is reported, related to the automatic creation of a test Lipoprotein Metabolism Ontology (LMO), whose terms were extracted automatically from 300 abstracts and then compared with a list of terms defined by human experts, showing a good overlapping.

Also based on ontologies is the paper by Coulet *et al*[21]. Authors investigate on the benefits of adopting bio-ontologies for guiding data selection during the preparation step for Knowledge Discovery in life sciences databases. A case study relative to the search of genotype-phenotype relationships in a familial hypercholesterolemia dataset is presented, with the objective of selecting genomic variants that modulate the disease, its symptoms or the metabolism and/or effect of a drug. The paper then shows how ontologies can effectively support the data selection task: this kind of demonstrations is almost lacking in the literature.

The tissue microarray database described by Viti *et al *[22] offers image sharing among users, ontological annotation of stored information, and integration of bioinformatics information from remote sources. The system enables users to annotate descriptions of uploaded images and analysis results by using MESH and Gene Ontology terms. This supports correlation studies between pathologies and biological processes. Authors show how the use of ontology terms makes it possible to easily retrieve scientific literature and to add pathology and bioinformatics data.

Another pillar of the Semantic Web are mediators, which make possible to individuate suitable resources. Navas-Delgado *et al *[23] present an ontology-based mediator infrastructure, developed in the context of the Amine System Project (ASP) [24], aiming at 3D structure homology modeling of polypeptides. The paper includes a proposal for a generic infrastructure for knowledge management on the Semantic Web that is based on two interrelated ontologies: Ontology Metadata Vocabulary and Semantic Directory Metadata Ontology (SDMO).

The paper by Splendiani [25] aims at bringing together Semantic Web technologies and the very hot research area of systems biology. The author presents RDFScape, a plug-in software for Cytoscape, a widely used tool for the visualization of biological interactions. RDFScape allows to visualize and, especially, to reason on ontologies by representing them as biological pathways. The common usage of ontologies in biology is limited to annotation purposes. Instead, their use for the interpretation of high-throughput biological data can benefit from knowledge inference, thus allowing to use ontologies as knowledge-bases from which new information can be derived. Two examples are presented, showing how ontologies can be visualized as interaction networks, and how reasoning can be implemented.

### Formal methods in Systems Biology

Formal methods, which are widely used in theoretical computer science to formally define and analyze complex software systems, have found a natural application in modelling and simulation of biological systems and processes. Even though a specific formal language able to describe all the properties of biological systems has not yet been designed, some properties of formal languages can be very useful to describe and manage some interesting aspects, like, e.g., managing stochastic behaviours and asking simulated systems some “logic questions”. Selected papers addresses one of these two aspects. Bracciali *et al *[26] introduce the development of a stochastic model for the simulation of synaptic processes in which interacting biomolecular entities are represented as interacting processes. Bodei *et al *[27] apply techniques from formal methods and computational logic to develop an abstract qualitative model of metabolic networks. By means of the tool they present, it is possible to determine causal dependencies amongst molecules involved in metabolism. Biochemical reactions are expressed in terms of logical implications and “what-if” gene-knockout experiments can be performed.

### Algorithms for Bioinformatics

A bridge between Systems Biology and the session on Algorithms in Bioinformatics is represented by the paper from Francesconi *et al *[28]. Authors propose a new method to infer pathways networks on the base of the statistical measurement of the significance of pathways intersections. The topology of a network is reconstructed according to gene expression measurement datasets.

The session on Algorithms for Bioinformatics was represented by other two contributions. The article by Ferro *et al *[29] introduces the GraphFind software, which implements an efficient graph searching algorithm together with advanced filtering techniques. Graphs naturally model bioinformatics data and their relationship, as well as biomolecular systems. Therefore, a key role is going to be played by systems able to search for exact or approximate occurrence of a query graph.

In the paper from Brunetti *et al *[30], authors present a parallel algorithm for efficiently solving the sequence tagging problem. De novo protein identification is actually one of the most challenging problem in proteomics.

### Network Tools and Applications in Biology

The session on the general theme (Network Tools and Applications in Biology) is here represented by two contributions. These were selected among five presentations that were submitted under the common subtitle “From components to processes” and were aimed at presenting results of the German HOBIT (Helmhotz Open Bioinformatics Technology) [31] project.

The paper from Margaria *et al *[32] introduces Bio-jETI, a platform for service integration, design and orchestration, dedicated to interdisciplinary work between computer scientists and biologists, which claims to allow biology domain experts, not trained in computer science, to directly define complex service orchestration and to use complex bioinformatics tools in a simple and intuitive way. Bio-jETI relies on a framework that has been used over some years in the telecommunication domain. In this paper, the framework is described together with some use cases in bioinformatics. Major strengths of the platform are its formal verification capability and remote tool integration.

An application of the Bio-jETI platform for service modeling and execution is presented in the paper by Lamprecht *et al *[33]. Authors describe a workflow developed by using the Bio-jETI platform with the aim of re-engineering GeneFisher, a popular tool for designing PCR primer for genes of unknown sequence on the basis of genes that are known to exist in another species. The paper includes considerations on turning a component-based application to a collection of composite services that implement complex processes and a discussion about the difference between data driven and control-flow based workflow models.

## Competing interests

The authors declare that they have no competing interests.
